# MALDI-TOF Mass Array Analysis of *Nell-1* Promoter Methylation Patterns in Human Gastric Cancer

**DOI:** 10.1155/2015/136941

**Published:** 2015-05-19

**Authors:** Changlu Gao, Qian Zhang, Deyang Kong, Di Wu, Changlei Su, Jinxue Tong, Feng Chen, Qifan Zhang

**Affiliations:** ^1^Department of Surgical Oncology, The Fourth Hospital of Harbin Medical University, Harbin 150010, China; ^2^Central Laboratory, School of Stomatology, Peking University, Beijing 100081, China; ^3^Department of Nephrology, First Affiliated Hospital of Harbin Medical University, Harbin 150010, China

## Abstract

Mass spectrometry (MS) enables rapid and sensitive qualitative and quantitative analyses of biomolecules (proteins, peptides, oligosaccharides, lipids, DNA, and RNA), drugs, and metabolites. MS has become an essential tool in modern biomedical research, including the analysis of DNA methylation. DNA methylation has been reported in many cancers, suggesting that it can be utilized as an early biomarker to improve the early diagnosis rate. Using matrix-assisted laser desorption/ionization time-of-flight MS and MassCLEAVE reagent, we compared *Nell-1* hypermethylation levels among tumor tissues, paracarcinoma tissues, and normal tissues from gastric cancer patients. Almost 80% of the CpG sites in the amplicons produced were covered by the analysis. Our results indicate a significant difference in methylation status between gastric cancer tissue (a higher level) and normal tissue. The same trend was identified in gastric cancer tissue versus paracarcinoma tissue. We also detected lower relative expression of *Nell-1* by real-time PCR. Furthermore, immunohistochemical analyses revealed that *Nell-1* staining was less intense in cancer tissue relative to normal tissue and that the tumor cells had spread to the muscle layer. These findings may serve as a guide for the early diagnosis of gastric cancer.

## 1. Introduction

Gastric cancer is one of the most common malignant tumors and the second leading cause of cancer-related deaths worldwide. Indeed, gastric cancer has one of the highest mortality rates of all cancer types and a high incidence in Asia [1]. Nearly three-quarters of cases occur in developing countries, and almost half of all cases occur in East Asia, mainly in China [2]. Because gastric cancer is an aggressive disease with nonspecific early symptoms, it is typically diagnosed only at an advanced stage [3, 4].

There are many established treatments for gastric cancer [3], of which surgery remains the most important for patients without major metastasis [5]. However, despite progress in surgical techniques, the overall 5-year survival rate for gastric cancer patients is low while the cost of treatment is high [6]. To improve the early diagnosis rate, the identification of biomarkers of gastric cancer is important [7]. Like other cancers, gastric cancer is a complex and heterogeneous disease. The development and progression of gastric cancer is associated with epigenetic mechanisms, especially DNA methylation [8, 9]. Epigenetic alterations such as changes in DNA methylation and histone acetylation/methylation are common in many cancers [10].

The best characterized mechanism is transcriptional silencing events, associated with hypermethylation of the promoter regions of genes that regulate important cell functions [11]. DNA methylation and related chromatin changes are important processes in the regulation of gene expression. Studies of various types of cancer have demonstrated the relevance of these regulatory changes. Promoter hypermethylation of tumor suppressor and tumor-related genes, including* APC*,* hMLH1*,* RUNX3*,* CACNA2D3*,* DKK-3*,* Cystatin*,* CBS*,* GPX3*, and* MYO1A*, has also been observed in gastric cancer [12–20]. Gene promoter hypermethylation has been examined by methylation-specific PCR (MSP) and bisulfite genomic sequencing (BGS) in human gastric cancer tissues.

The* Nell-1* gene has been mapped to chromosome 11p15 [21]; the high prevalence of* Nell-1* promoter methylation in colon cancer suggests a role for inactivation of the gene in colon tumorigenesis. Additionally,* Nell-1* is a candidate tumor suppressor gene [22].* Nell-1* promoter hypermethylation is a common, tissue-specific event in human esophageal adenocarcinoma (EAC), and it is a potential biomarker of a poor prognosis in early-stage EAC [23]. Based on these findings, we hypothesized that* Nell-1* is inactivated via promoter hypermethylation in human gastric cancer and that this modification may be used as a biomarker for early detection of the disease.

Mass spectrometry (MS) can discriminate between methylated and nonmethylated samples and identify differentially methylated sites with rapidness and accuracy. Matrix-assisted laser desorption/ionization time-of-flight (MALDI-TOF) MS and the MassCLEAVE reagent (Sequenom, San Diego, CA) are used for the high-throughput quantitative analysis of DNA methylation status in the Sequenom EpiTYPER assay [24, 25]. The utility of this method for quantifying methylated and unmethylated DNA molecules was confirmed by the Sequenom group [26].

To assess whether* Nell-1* is inactivated via promoter hypermethylation in human gastric cancer, we investigated the methylation status of the* Nell-1* promoter in 75 samples from 25 patients using MALDI-TOF MS. Our results show that* Nell-1* promoter hypermethylation is a common event in gastric cancer.

## 2. Materials and Methods

### 2.1. Sample Collection

In the current study, 25 gastric cancer tissues, 25 paracarcinoma tissues (3 cm from the tumor margin), 25 normal tissues, and 25 formalin-fixed paraffin wax-embedded gastric cancer tissues and normal tissues were collected from 25 gastric cancer patients who were diagnosed and underwent surgery at the Fourth Affiliated Hospital, Harbin Medical University (Harbin, China), between April and September of 2012. The patients' characteristics are shown in Table 1. All primary gastric cancers were evaluated according to the criteria set forth in the 7th edition of the American Joint Committee on Cancer Staging Manual. The diagnosis of primary gastric cancer was confirmed by hematoxylin and eosin (H&E) staining. The “normal” gastric tissues in this study were histologically verified to have no neoplastic changes.

Fresh tissue samples were obtained during resection surgery, immediately snap-frozen in liquid nitrogen, and stored at −80°C until they were used for DNA extraction. In this study, none of the patients received chemotherapy or radiation therapy before surgery. The primary gastric cancer tissue, tissue adjacent to the gastric cancer, and normal tissue from each patient were allocated to the gastric, paracarcinoma, and normal groups, respectively.

All participants provided informed consent prior to their participation in the study. All procedures were approved by the ethics committees of the hospitals involved.

### 2.2. Immunohistochemistry (IHC) and H&E Staining

Formalin-fixed, paraffin wax-embedded gastric cancer tissues and normal tissues were used for IHC and H&E staining. For IHC, tissues were fixed in 10% methanol/buffered formalin for 12 h and were processed routinely. The paraffin wax-embedded sections (4 *μ*m) were immersed in xylene and rehydrated through an ethanol series. Endogenous peroxidase activity was blocked by incubating the sections in 3% H_2_O_2_ for 15 min. After washing with phosphate-buffered saline (PBS), the sections were subsequently submerged in EDTA (pH 9) and exposed to microwave heating for antigen retrieval, followed by 3 min of treatment in a prewarmed pressure cooker with antigen retrieval citrate buffer (pH 6.8). Following depressurization, cold water was poured into the cooker for 10 min. The sections were then washed three times with PBS and incubated in goat serum for 20 min. The slides were immersed in anti-*Nell-1* rabbit polyclonal antibodies for 1 h. After washing with PBS, the slides were incubated with horseradish peroxidase-conjugated anti-rabbit antibodies for 1 h. The slides were then examined under a microscope.

### 2.3. DNA Extraction

For DNA extraction, frozen tissues were obtained and reviewed to confirm that the tumor content was >80% of the section area. DNA was extracted from the frozen tissues using a QIAamp DNA Mini Kit (Qiagen AG, Basel, Switzerland) following the manufacturer's protocol; the DNA samples were dissolved in a final volume of 100 *μ*L. The quantity of DNA (A260/A280) in each sample was measured using a NanoDrop 2100 spectrophotometer before sequencing (Thermo Fisher Scientific, Waltham, MA).

### 2.4. Real-Time PCR Analysis

Total RNA was extracted from normal and gastric cancer tissues using TRIZOL reagent (Invitrogen, Carlsbad, CA). Agarose gel electrophoresis and a NanoDrop 2000 spectrophotometer (Thermo Fisher Scientific) were employed to assess the quality and concentration of RNA. A total of 1 *μ*g of high-quality RNA was reverse-transcribed to first-strand cDNA with Superscript First-Strand Synthesis SuperMix (Invitrogen). Real-time PCR was conducted with SYBR Green Master mix (Applied Biosystems, Foster City, CA). The reaction (95°C for 15 s and 60°C for 1 min) was run using the ABI PRISM 7500 Real-Time PCR System (Applied Biosystems) with relative expression analyzed by the 2^−ΔΔCt^ method. The sequences of the primers used to amplify* Nell-1* and the control gene* GAPDH* are listed in Table 2.

### 2.5. Primer Design and PCR Using the Sequenom MassARRAY System

**Figure 1:**
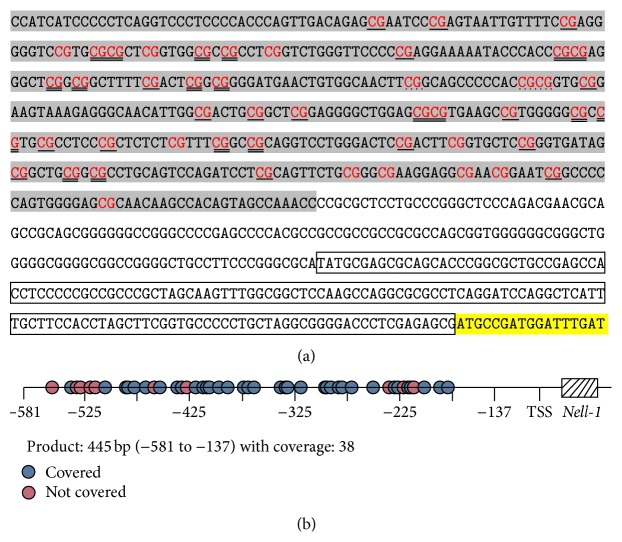
Amplicon size and locations of informative CpG sites in amplicons of the* Nell-1* gene. (a) The sequences of targeted gene. The amplified region was colored in gray. Transcription start sequences were labeled in frame and transcription sites were labeled in yellow. CpG sites in amplicons appear in red and single line represented separate detection. Double line showed testing with the adjacent site. Point line showed adjacent three-site testing. No line showed that CpG sites cannot be detected in amplified sequences. (b) The amplicon map and the locations of CpG sites.

The primers designed for* Nell-1* covered those regions with the most CpG sites. Most selected amplicons were located in the promoter region, from −581 to −137, relative to the transcription start site (Figure 1). The primers were designed using EpiDesigner [18]. For amplification, the reverse primer had a T7-promoter tag added, and the forward primer had a 10-mer tag sequence added to balance the PCR primer lengths (F: aaagagagGGTTTGGTTATTGTGGTTTGTTG and R: cagtaatacgactcactatagggagaaggctAACCATCATCCCCCTCAAAT). Genomic DNA was treated with bisulfite using an EpiTect Bisulfite Kit (Qiagen AG). The bisulfite-treated genomic DNA was then amplified under the following conditions: initial denaturation, 94°C for 4 min, then 45 cycles of 94°C for 20 s, 56°C for 30 s, and 72°C for 1 min, followed by 3 min at 72°C. The products were stored at 4°C for further analysis.

### 2.6. *In Vitro* Transcription and T-Cleavage (RNase A Digestion) Assay

Shrimp alkaline phosphatase (SAP; Sequenom) was used to remove unincorporated dinucleotide triphosphates (dNTPs). The components were 1.7 *μ*L of RNase-free ddH_2_O and 0.3 *μ*L of SAP combined with the above PCR products. The mixture was incubated at 37°C for 20 min, 85°C for 5 min, and then at 4°C indefinitely. Using T7 R&DNA polymerase (Epicentre, Madison, WI), thymidine triphosphate was incorporated into the PCR product, finishing the transcription reaction. Ribonucleotides and dNTPs were used at concentrations of 1 and 2.5 mmol/L, respectively. RNase A (Sequenom) was added to the same reaction to cleave the transcripts (T-cleavage assay). The reaction mixture was incubated at 37°C for 3 h. To remove the phosphate backbone, the T-cleavage/RNase A assay reaction products were diluted with 20 *μ*L of RNase-free H_2_O and mixed with Clean Resin (Sequenom) before performing MS.

### 2.7. Mass Spectrometry

The entire RNase A and clean resin-treated product was robotically dispensed onto silicon matrix preloaded chips (SpectroCHIP; Sequenom) and mass spectra were collected using a MassARRAY Compact MALDI-TOF Analyzer (Sequenom). The mass spectra methylation ratios were generated using EpiTYPER (ver. 4.0; Sequenom).

### 2.8. Statistical Methods

The data were analyzed using the SPSS software (ver. 17 for Windows; IBM, Armonk, NY). An analysis of variance (ANOVA) was used to identify significant differences in methylation rates among the samples. All *P* values were two-sided; *P* < 0.05 was considered to indicate statistical significance.

## 3. Results and Discussion

Increasing numbers of cancer deaths are projected to occur in Asia; this trend can be combatted by expanding knowledge of cancer control and the underlying genetic mechanisms [2]. Although much has been learned about the genetic causes of cancer, the details remain unclear [27].

In a previous study, MSP and BGS were used to analyze promoter methylation [28]; however, these methods have similar disadvantages. For this reason, Sequenom created a high-throughput quantitative assay for DNA methylation using MALDI-TOF MS and MassCLEAVE reagent. This method is highly accurate with regard to specific methylation sites, and it enables both quantitative assessment of methylation levels and analysis of multiple methylation sites [16, 24, 25, 29].

In this study, we analyzed the methylation patterns of the* Nell-1* gene in 25 gastric cancer tissue samples, 25 tissues adjacent to gastric cancer, and 25 normal samples. The* Nell-1* sequences analyzed contained CpG-rich regions. In total, 48 CpG sites per sample were analyzed. More than 50% of the CpG sites in* Nell-1* were detected in amplicons, and almost 80% of the CpG sites in the amplicons were used in our analysis (Table 3).

**Figure 2:**
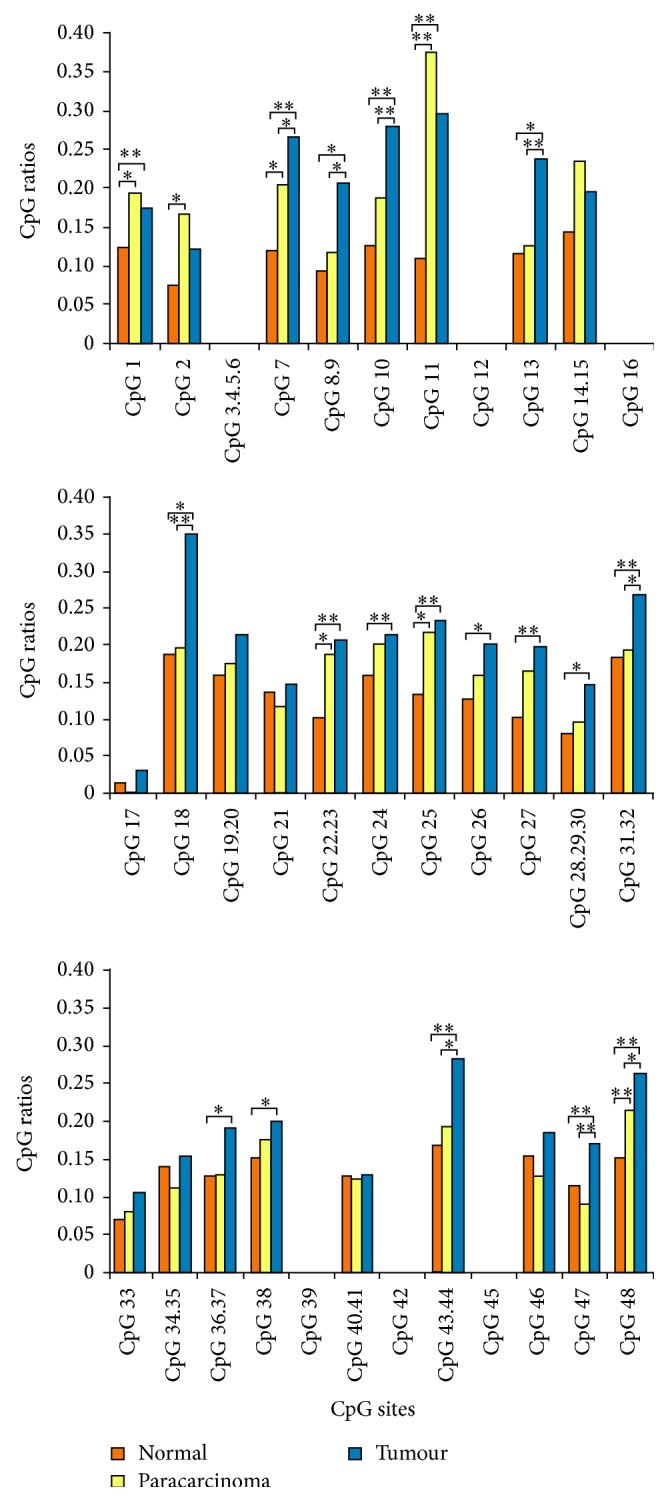
CpG ratios of CpG sites in the* Nell-1* gene covered and not covered by MALDI-TOF MS analysis. Error bars and comparisons among tumour tissues, paracarcinoma tissues, and normal tissues are shown.

The three types of samples showed different methylation levels at the various CpG sites in* Nell-1*. An ANOVA demonstrated significant differences in the methylation levels of* Nell-1* between the gastric cancer and normal samples (*P* < 0.05 at 8 CpG sites and *P* < 0.01 at 11 CpG sites). Additionally, significant differences were detected between the gastric cancer and paracarcinoma samples (*P* < 0.05 at five CpG sites and *P* < 0.01 at four CpG sites). However, there was no significant difference between the paracarcinoma and normal samples (eight CpG sites with *P* < 0.05). Also, the gastric cancer samples had higher degrees of methylation relative to the normal samples (Figure 2). The abnormal methylation of DNA is an early event in carcinogenesis and can serve as a biomarker for early detection [30]. In this study, we demonstrated that the methylation level of* Nell-1* is an important issue in gastric cancer. This finding may be useful in the earliest stages of clinical disease. However, before considering the methylation status of the* Nell-1* promoter as a biomarker in gastric cancer and normal tissues, further research is needed. In cancer patients, promoter hypermethylation has some prognostic value. It has been reported that* Nell-1* binds to a specific membrane protein (APR3) to mediate the downregulation of cyclin D1 [31]. Based on a functional analysis of cell proliferation and osteoblast differentiation, APR3 binding may be the mechanism by which* Nell-1* promotes osteoblast differentiation [32]. Thus,* Nell-1*, as a suppressor gene may play a role in cancer metastasis.

**Figure 3:**
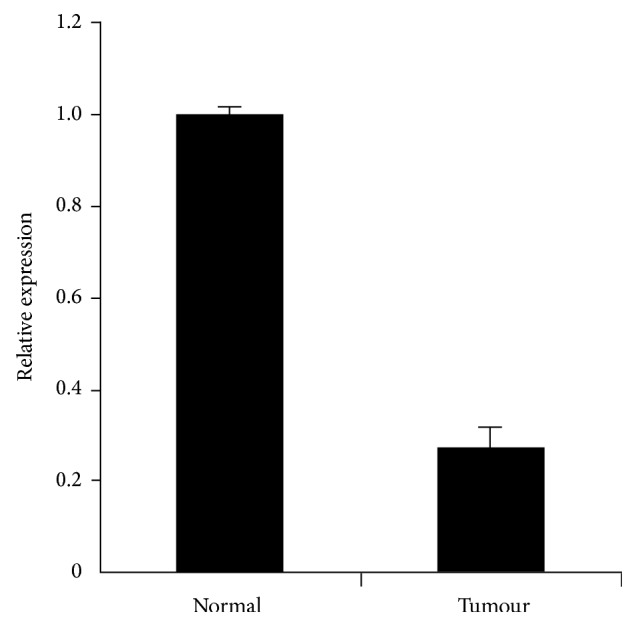
*Nell-1* mRNA expression analysis. Quantitative polymerase chain reaction (qPCR) was performed to identify the mRNA expression of* Nell-1* in normal tissues and tumour tissues.

**Figure 4:**
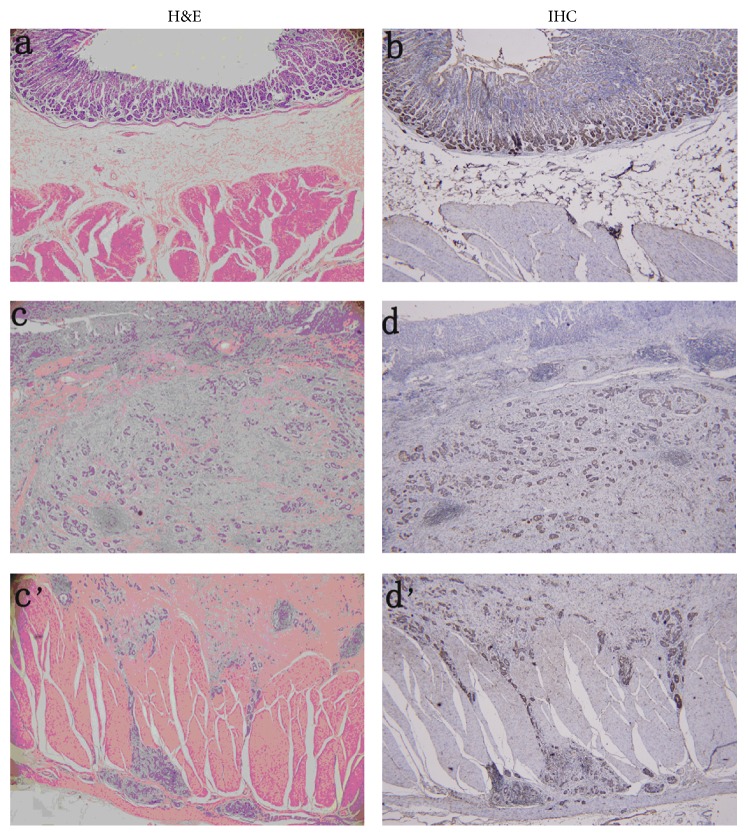
Immunohistochemistry and hematoxylin and eosin staining of* Nell-1* protein expression (a, b) normal tissues; (c, d) mucosal layer of tumour tissues; (c', d') muscular layer of tumour tissues.

We also found that the transcription and translation of* Nell-1* were decreased in the gastric cancer samples compared to the normal samples. This may be explained by the fact that the methylation of a gene can reduce its expression. Quantitative PCR showed that the mRNA expression of* Nell-1* in the gastric cancer tissues was decreased threefold compared with that in the normal tissues (Figure 3). As expected, immunohistochemical analyses of gastric cancer samples showed a lower intensity of* Nell-1* staining and that the cancer cells had spread to the muscle layer (Figure 4). The malignant transformation of gastric cells occurs via a multistep process involving various genetic and epigenetic changes [33]. The relationship between* Nell-1* methylation and* Nell-1* protein expression requires further confirmation. Transcriptional silencing of the* Nell-1* promoter is an early and common event in human EAC [23] and colon tumorigenesis [22]. Increased methylation of* Nell-1* may play a role in other cancers [34]. Our results indicate that the methylation level of the* Nell-1* promoter is related to the spread of gastric cancer cells.* Nell-1* expression was detected in normal and gastric cancer tissues, suggesting a physiological role in normal gastric tissues. It will be interesting to investigate the correlation of* Nell-1* promoter methylation with the transformation of gastric cancer cells and cancer prevention.

## 4. Conclusions

MALDI-TOF MS is a useful tool for the analysis of DNA methylation. Methylation of the* Nell-1* promoter is a common event in gastric cancer compared with normal tissue.* Nell-1* promoter methylation reduced the expression of* Nell-1* in gastric cancer tissues, as determined by a lower intensity of IHC staining, with expansion to the muscle layer. This suggests that* Nell-1* can be used as a biomarker for the diagnosis of gastric cancer.

## Figures and Tables

**Table 1:** The clinical characteristics of study subjects.

GC^a^	Gender	Age (year)	Differentiation	AJCC^b^ stage
GC01	Male	76	Poor	III
GC02	Female	59	Poor	I
GC03	Male	60	Moderate/poor	III
GC04	Male	48	Poor	III
GC05	Male	77	Moderate	II
GC06	Female	69	Poor/mucinous	III
GC07	Male	49	Poor/mucinous	III
GC08	Female	58	Moderate/poor	II
GC09	Male	54	Poor	I
GC10	Female	70	Moderate/poor	III
GC11	Male	52	Moderate	II
GC12	Male	57	Poor/mucinous	III
GC13	Female	52	Poor/mucinous	II
GC14	Male	70	Moderate/poor	II
GC15	Male	65	Moderate/poor	III
GC16	Male	58	Poor	II
GC17	Male	58	Moderate/poor	II
GC18	Female	71	Poor	III
GC19	Female	55	Poor	III
GC20	Female	58	Poor	III
GC21	Female	64	Poor	III
GC22	Male	71	Moderate	I
GC23	Male	50	Poor/mucinous	III
GC24	Female	76	Moderate/poor	III
GC25	Female	55	Moderate	I

^a^GC: gastric cancer; ^b^AJCC: the American Joint Committee on Cancer.

**Table 2:** The primer sequences of *Nell-1* and GAPDH genes.

Human gene	Forward primer sequence (5′ → 3′)	Reverse primer sequence (5′ → 3′)
*Nell-1 *	GCTTTGGGATGGACCCTGAC	GAAATAAAAATGCTTTGCTGGC
GAPDH	GTCTCCTCTGACTTCAACAGCG	ACCACCCTGTTGCTGTAGCC

**Table 3:** MALDI-TOF MS analysis of CpG sites in amplicons of the *Nell-1* gene.

Gene	Amplicon size (bp)	Total number of CpG sites in amplicon	Number of analyzed CpG sites in amplicon	Number of analyzed CpG sites in amplicon	
				Single sites	Composite sites
*Nell-1 *	445	48	38	17	21

## Abbreviations

MALDI-TOF MS: Matrix-assisted laser desorption/ionization time-of-flight mass spectrometry
IHC: Immunohistochemistry
H&E: Hematoxylin and eosin.
